# Difficulty of the ethical decision–making process in withholding and withdrawing life-sustaining treatments in French EDs during COVID pandemic

**DOI:** 10.1186/s13049-020-00772-3

**Published:** 2020-08-13

**Authors:** Marion Douplat, Laurent Jacquin, Soizic Frugier, Karim Tazarourte, Pierre Le Coz

**Affiliations:** 1 Emergency Department, Lyon Sud Hospital, University Hospital, Hospices Civiles of Lyon, 165 chemin du Grand Revoyet, F-69495 Pierre Bénite, France; 2 UMR ADéS 7268, Aix-Marseille University/ EFS / CNRS, Espace éthique méditerranéen, Timone Adulte’s Hospital, Marseille, France; 3 Emergency Department, Édouard-Herriot Hospital, Lyon University Hospital, Hospices Civiles of Lyon, 5 place d’Arsonval, F-69003 Lyon, France

France is one of the most impacted countries in the world by the COVID-19 pandemic, with more than 170,000 confirmed cases. Thus, one of the ethical dilemmas faced by French emergency physicians concerns the decision of withholding or withdrawing life-sustaining treatments, especially given the impact of the COVID-19. What’s more, we have already shown, prior to the outbreak of COVID-19, that the decision-making process of these decisions is especially difficult in the context of emergency medicine because of the lack of time and the absence of anticipation in chronic diseases [1]. In the context of COVID-19, healthcare teams face several challenges with the decisions to withhold or withdraw life-sustaining treatments.

The first challenge is the fair allocation of medical resources depending on the area of France. Indeed, the Great East region, which was hit first by the pandemic, was the most impacted because of a shortage of critical care beds. The other regions were able to get organized in time and increased their ICU bed capacity. Several recommendations have since been proposed, setting the respect of ethical values and the necessity to treat people equally as a priority [2].

The second challenge is the communication between healthcare teams and relatives. We showed that healthcare teams expressed difficult experiences regarding the announcement and the communication with the patients and their relatives concerning these decisions [3]. Moreover, we found that relatives suffered after a decision of withdrawing or withholding life-sustaining treatments and displayed symptoms of anxiety and depression, which persisted over time [4]. During the COVID-19 pandemic, the relatives were not physically present in EDs because of the ban on hospital visits. Therefore, lots of announcements were made by phone and may have induced additional trauma, given that relatives were not able to accompany their loved ones.

The third challenge we identified is the impact of these decisions on the healthcare teams. We found in a previous study that the decision-making process to withhold or withdraw life-sustaining treatments often triggers a feeling of isolation and being overwhelmed among ED physicians. Mental health issues have been reported among healthcare workers, especially for those who were directly engaged in the care of patients with COVID-19 [5], but no data was available concerning the impact of the decisions to withhold or withdraw life-sustaining treatments. Training healthcare teams to develop an effective communication strategy in the context of a pandemic is a fundamental step in preventing stress disorders, and must be evaluated for improvement.
